# What Time Alone Offers: Narratives of Solitude From Adolescence to Older Adulthood

**DOI:** 10.3389/fpsyg.2021.714518

**Published:** 2021-11-01

**Authors:** Netta Weinstein, Thuy-vy Nguyen, Heather Hansen

**Affiliations:** ^1^School of Psychology and Clinical Language Science, University of Reading, Reading, United Kingdom; ^2^Department of Psychology, Durham University, Durham, United Kingdom

**Keywords:** solitude, self-determination theory, autonomy, aging, well-being, adolescence, loneliness

## Abstract

Solitude – the state of being alone and not physically with another – can be rewarding. The present research explored the potential benefits of solitude from a pragmatist approach: a ground-up, top-down perspective that is receptive to new knowledge but informed by theory. Participant recruitment was stratified by age and gender, and the sample involved 2,035 individuals including adolescents (13–16 years), adults (35–55 years), or older adults (65+ years). Data were analyzed with a mixed-methods approach. Coded themes from brief narratives about solitude were extracted, and their frequencies (i.e., *their salience to participants*) were compared across the lifespan. Themes were then correlated with two indicators of well-being in solitude: self-determined motivation for solitude and peaceful mood. Several prominent themes emerged when talking about time spent in solitude. With the exception of feeling competent in solitude, which was described frequently but consistently unrelated to self-reported well-being regardless of age, benefits of solitude tended to shift over the lifespan. Some qualities, such as a sense of autonomy (self-connection and reliance; absence of pressure), were salient and consequential for everyone, but increasingly so from adolescence to older adulthood. Older adults also reported feeling most peaceful in solitude and described their social connection and alienation less frequently, suggesting they see solitude and social time as more distinct states. Findings are discussed in light of existing work on solitude across the lifespan, and theoretical frameworks that spoke well to the data (e.g., self-determination theory).

## Narratives of Solitude: a Lifespan Perspective on What is Learned and What is Gained

Solitude – the state of being alone and not physically with another (Nguyen et al., 2021) – is increasingly understood to confer benefits. Until now, research in social, developmental, and clinical psychology, and in medicine, has mainly examined drawbacks of solitude and certain negative psychological states broadly associated with it, such as loneliness and social anxiety (e.g., Heinrich and Gullone, 2006; Cacioppo and Hawkley, 2009; Hawkley and Cacioppo, 2010; Coplan et al., 2015). But a growing body of work focuses on positive solitude by identifying and classifying the ways in which people flourish when alone. It is now quite clear that solitude is distinct from loneliness, the feeling of alienation from others (Galanaki, 2004, 2005), and isolation, the experience of choiceless and extended alone time (Coplan and Bowker, 2013). Compared to the vast knowledge researchers have about loneliness and alienation, positive solitude research is nascent and additional work is needed to identify and describe potential benefits.

The current research explored content within brief written narratives of “everyday” solitude. The primary interest was in the lessons learned, and benefits of, time spent alone, because less is known about these aspects of solitude. However, to avoid biasing participants toward an unduly positive view of solitude that would lead them to describe benefits they would not otherwise endorse, instructions asked about both benefits and costs. Furthermore, the study examined whether and how these qualities of solitude change across the lifespan, from adolescence to adulthood, because previous work suggests the characteristics and challenges associated with developmental stages affect people’s experiences in solitude (Coplan and Bowker, 2013).

The focus was on time spent in solitude during 3 months of the COVID-19 outbreak, which took place during the time of this research. During this time, solitude was presumed to be challenging and individuals spent more time in solitude than they had before. Despite this increased exposure to solitude, few emotional costs were identified as a direct function of spending time in solitude during these early months (Weinstein and Nguyen, 2020). At the same time, some learned to appreciate solitude during this time (Peyser, 2021). The present research was designed to capture what was learned about time spent alone.

### Solitude Across the Lifespan

Multiple studies point to the importance of taking a lifespan perspective in understanding solitude. From adolescence (Thomas and Azmitia, 2019), onward to adulthood and older adulthood (Long and Averill, 2003; Ost Mor et al., 2020), individuals experience solitude as a positive space rich in opportunity (Buchholz and Catton, 1999; Long et al., 2003). Researchers are beginning to index and thus better understand the benefits of solitude (also referred to as positive solitude) by developing comprehensive explanations of what can be gained from time spent alone. Those benefits can either be aimed inward (directed internally) or outward (directed toward others and connections in the world; Long et al., 2003).

Very recently, qualitative work was conducted which involved interviews with older adults and their adult caregivers (Ost Mor et al., 2020) about the positive experience of “being with myself.” Interviews revealed a number of benefits of solitude and observed that they varied across the span of adulthood. In particular, as compared to mid-life adults, older adults described more quietness and interface with nature, recreation, hobbies, or habits. Similarly, in other work, older adults appeared to be better than younger people at using their alone time to reap benefits for their well-being (Larson, 1990), including benefits to positive emotions (Lay et al., 2019) and emotion regulation (Rokach and Brock, 1998).

Solitude benefits may evolve early in life, with benefits readily increasing from adolescence through older adulthood. While pre-adolescents (aged 11 and 12) may struggle with the process of separating from their parents and thus experience anxiety during time spent alone, middle adolescents have expressed they increasingly appreciate time alone (Larson, 1997), and throughout the teen years adolescents have a more positive attitude toward aloneness (Marcoen et al., 1987; Corsano et al., 2006). It has been proposed that as adolescents age, time alone becomes a space for freedom (i.e., from parental influences), reflection, and creativity (Ammaniti et al., 1988).

### Assessing Well-Being in Solitude

Self-determination theory (SDT; Ryan and Deci, 2000a,b; Deci and Ryan, 2008a; Ryan and Deci, 2017) provides a useful framework for understanding well-being in solitude along the lifespan. SDT-informed research posits that solitude is more beneficial to the extent that it is undertaken for self-determined motivation (i.e., driving reasons) coming from one’s own interests and values rather than from internal demands or pressures (Nguyen et al., 2018; Thomas and Azmitia, 2019). In the context of solitude, self-determined motivation is an important aspect of solitude in both adulthood and older adulthood (Ost Mor et al., 2020). Arguably, this aspect of being alone is sufficiently important in that it defines positive solitude and differentiates it from isolation (Burger, 1995). Furthermore, motivation for solitude may change throughout development just as the benefits of solitude do. For example, older adults are more likely to choose time alone (Larson, 1990), and self-determined solitude is associated with more positive emotions in older adults (Lay et al., 2020). Still, the question of how self-determined motivation for solitude relates to beneficial experiences across the lifespan remains largely unanswered.

Well-being in solitude is also experienced through peaceful (i.e., relaxed and not lonely) affect. Loneliness is associated with detrimental solitude (or self-isolation) and has substantial implications for mental and physical health (Victor et al., 2000; Matias et al., 2011; O'Súilleabháin et al., 2019; Smith and Victor, 2019). But loneliness is not isomorphic with the state of solitude. Instead, it can be understood in terms of its associations with cognitive perceptions of one’s alone and social space (Hawkley and Cacioppo, 2010; Russell et al., 2012; Goossens, 2018) and can also be experienced when around others (Van Baarsen et al., 2001). Individuals may feel well-being benefits of their solitude in other ways than the absence of loneliness. Feeling relaxed is perhaps the most noteworthy of emotional well-being benefits (Buchholz, 1997). For example, Nguyen et al. (2018) identified that most of the enjoyment in solitude comes in the form of low-arousal positive affect, namely, relaxation. Similarly, Ost Mor et al. (2020) identified that positive solitude is enjoyable in no small part because it is relaxing.

### Current Research

*Salience* of solitude benefits can be explored by asking participants to reflect on what they learn and how they benefit from their time alone. Salient benefits should be spontaneously described with greater frequency. However, to understand the *significance* (e.g., for well-being) of those themes, it is useful to evaluate how they relate to separate perceptions of well-being during solitude. There has been little research documenting the gains of solitude across the adolescent to older adult lifespan. The current research was intended to build understanding of potential gains made in solitude, while staying open to the potential costs of being alone. Brief narratives of solitude were examined over 3months, through a pragmatist approach that sought to maximize both insights from data and from theory (Howe, 1988; Onwuegbuzie and Johnson, 2006; Creswell and Plano Clark, 2011).

The pragmatist perspective reflected two study goals: theory building without prior assumptions and conferring new theoretical perspectives to relate back to the existing literature on solitude. To do so, the study used a qualitative analytic method, namely, thematic analysis, strategically: First, themes were formulated while staying naïve to existing theory, and then, themes were refined and tested applying expert perspectives to the data. Findings were also validated through quantitative analyses relating themes to well-being in solitude. This was in line with the current research goals (Johnson and Onwuegbuzie, 2004) of evaluating salience of solitude benefits as well as their significance.

To summarize, a data-driven approach was used to draw out, identify, and code themes without particular bias from previous work in any particular age group. Then, the personal significance of those identified themes was tested top-down, based on previous research and theory (Ryan and Deci, 2017; Nguyen et al., 2018).

Methodological and analytic decisions were registered in advance.^1^ The study tested pre-registered research questions (RQ):

1. What can be learned or gained from solitude? (*Qualitative*)
2. Are these benefits (of RQ1) associated with self-determined motivation for solitude? (*Quantitative*^2^)
3. Are these benefits (of RQ1) associated with two types of peaceful mood – relaxation and the absence of loneliness – in solitude? (*Quantitative*)

## Materials and Methods

### Study Recruitment and Sampling

This study analyzed data from three large purposive samples selected on the basis of age, gender, and a broad geographic spread within the United Kingdom. A first, adolescent sample consisted of *n*=1,001 13–16-year olds [534 (53.3%) boys, 464 (46.3%) girls, and 3 (0.4%) subjects who identified as another gender]. Adult participants were *n*=523 individuals between 25 and 51 years of age,^3^ who identified as male (250 or 47.8%), female (272 or 52.0%), or other (1 or 0.2%). Finally, older adults were comprised of *n*=511 individuals [215 men (42.1%) and 296 (57.9%) women] between the ages of 59–85 years.^4^ Three adults who did not give their age but were recruited with the adults were coded into the adult group.

Adolescents were recruited through ICM Unlimited, a social research polling company.^5^ No hard quota controls were set for this research. However, soft quotas were set to ensure a good spread of respondents of each age (13-, 14-, 15-, vs. 16-year olds), gender, and region across the United Kingdom. ICM Unlimited contacted parents first with an invitation email containing the link to the survey, targeted by relevant variables. The first part of the survey was targeted at adults of an appropriate age range (c 30–50). Parents were asked whether they had children of the relevant age group (13–16-year olds) and asked for consent for their child to take part in the research after being shown an information sheet and consent form designed to inform and support the agency of parents. Children then saw the information sheet and asked for their consent to take part in the survey with age-appropriate language. Parents left the room, and adolescents completed the online study. Adults and older adults were recruited through stratification on Prolific Academic, an online panel company. Recruitment was stratified by age ranges to represent adults mid-life, who could be clearly distinguished from adolescents, and older adults. This study received approval from the University of Reading School of Psychology Ethics Committee (Num. 2020-080-NW). All participants were paid for taking part.

### Narratives of Solitude

A diverse set of prompts was used to approach the issue of solitude benefits through a number of complementary lenses. Participants responded to two questions: “Over the past 3months many of us have spent quite a bit of time alone or without face-to-face contact with friends and family due to the coronavirus. During this time people have experienced many feelings, including happy, sad, lonely, and feeling relaxed. We are interested in hearing your unique experiences during this time, giving as much detail as you feel comfortable. Please tell us in a few sentences or in as much detail as you would like to give, as if you are writing in a journal.” This three-form design maximizes data resources to address complex issues with efficiency (Graham et al., 2006). Given the lead, “Think about times when you may have been on your own or alone in the past 3months,” participants were then randomly assigned to receive one of the following three prompts that asked them to think on the issue more deeply: “What have you learned?,” “What have you learned about yourself?,” or “What have you learned about the relationships in your life (for example with family, friends)?.” All questions also asked, “How have you learnt this?” All participants then received a second prompt using the same solitude instructions: “…What has made times spent being on your own, or alone, good or bad in the past 3months? Think about a time when it was especially easy or hard.”

### Measures of Positive Solitude

To ensure participants were thinking about time spent in solitude, the following measures all started with instructions to think back to “a time when you may have been on your own or alone.” Participants responded to the following items using a scale ranging from 1 (*does not apply to me at all*) to 7 (*applies to me very much*).

#### Self-Determined Motivation for Solitude

Self-determined motivation for solitude was measured with three items assessing: “Having time to myself was important and beneficial to me,” “I really valued having time by myself,” and “I felt I have no choice but to be alone,” taken from Nguyen et al. (2018). Reliabilities were as: *α*=0.57 for adolescents; *α*=0.63 for adults, and *α*=0.73 for older adults.^6^

#### Peaceful Mood Within Solitude

Relaxed and lonely moods were measured with five items: Relaxed mood was averaged from four items (calm, relaxed, at ease, and restful), and loneliness was measured with a single face-valid item (lonely). The average score of the four items assessing relaxed mood was further averaged with the reversed score for loneliness to reflect equal parts of both in a final mood measure. The two constructs were correlated moderately to highly: *r*=−0.44 in adolescents, *r*=−0.50 in adults, and *r*=−0.55, *ps*<0.001 in older adults.

### Data Processing and Analyses

#### Generating Narrative Codes

Themes were generated based on explorations of the raw data by five independent research assistants (and none of the authors) who were instructed on the basic concept that solitude can hold both costs and benefits. For practical considerations, adolescent data were considered first, and then, the group of five research assistants identified additional codes for themes unique to adult and older adult data. No analyses were conducted until codes had been set for the full sample. Three extensive conversations were held to discuss potential themes. These were moderated by the first author, whose job it was to minimize redundancy in different codes, and to establish both a conceptual clarity of codes and a common definition for each code agreed on by the research assistants.

Thus, decisions were made as a group to optimize each code for the following qualities: (1) comprehensiveness of data captured, (2) redundancy reduction, and (3) conceptual clarity. At this point, placeholder names were given to each code as a structure for undertaking the actual coding of the data. In most cases, identical themes observed across age groups allowed comparisons between adolescent, adult, and older adult data. But, in some cases, adults also raised themes not described by adolescents. In those cases, with respect to some narrative codes, only adult and older adult data could be compared.

#### Coding Narratives

Following the process of identifying codes, naïve researchers responsible for this task read through responses and coded narratives in terms of whether they had a theme present (coded 1) or absent (coded 0). Two researchers independently coded each data point, and a third independently coded a random subset. Following disagreements, researchers discussed the full set of codes and agreed on each theme for each response. Researchers were also given freedom to retain original codes if they did not see eye-to-eye. Final interrater agreement was *kappa*>0.80 for all codes. As pre-registered, to generate the final coding structure, a narrative was coded if any researcher identified the theme in that particular narrative.

#### Code Parsimony

The five researchers who determined the coding scheme identified a large number of codes (up to 25 codes after combining lowest order codes the researchers had initially agreed represented a single construct). A multi-step process was undertaken to identify higher order themes and to achieve greater code parsimony. First, the lead author of this paper considered codes in light of current theoretical approaches and literature in solitude reviewed above (namely, classification of solitude benefits by Long et al., 2003; self-determination theory, Ryan and Deci, 2017), as well as in light of additional insights from the data themselves, and generated 10 potential overarching categories that define the specific themes (i.e., codes). However, rather than sorting specific codes into their overarching themes herself, she asked experts in solitude (including two co-authors of this paper who were not actively involved in the coding process) to sort codes into the overarching theme categories or to indicate when a code did not fit into any of them. In this way, the overarching themes, and the placement of subthemes within them, were validated through multiple perspectives.

#### Expert Ratings

Experts (*M* years studying solitude=15.29; *SD*=11.28) reported they had studied adolescents (*n*=3), adults (*n*=6), and older adults (*n*=2) in relation to solitude. Each was asked to drag individual codes into their ideal overarching theme, or alternatively to identify if any code did not fit any of the proposed themes well. Specific codes were distributed into a theme when a majority vote of the eight experts placed them in the category (See supplemental materials^7^ for codes and their themes).

#### Exploratory Factor Analyses

Expert ratings were used to reduce the full set of codes to a smaller number of overarching themes. To ensure those themes were orthogonal, they were then subjected to an exploratory factor analysis. Specifically, a polychoric correlation matrix was conducted, which is similar to an exploratory factor analysis but more appropriate for dichotomous data (Holgado-Tello et al., 2010). Ten dimensions were requested (one for each variable) and a cutoff above 0.50 was used to determine whether items loaded together. The result showed that “autonomy” and “self-connection” loaded together (0.65 and 0.71), and indicated a meaningful covariation between “competence” (0.55) and “activities” (0.61). Other variables did not load together.

#### Final Set of Themes

**Figure 1:**
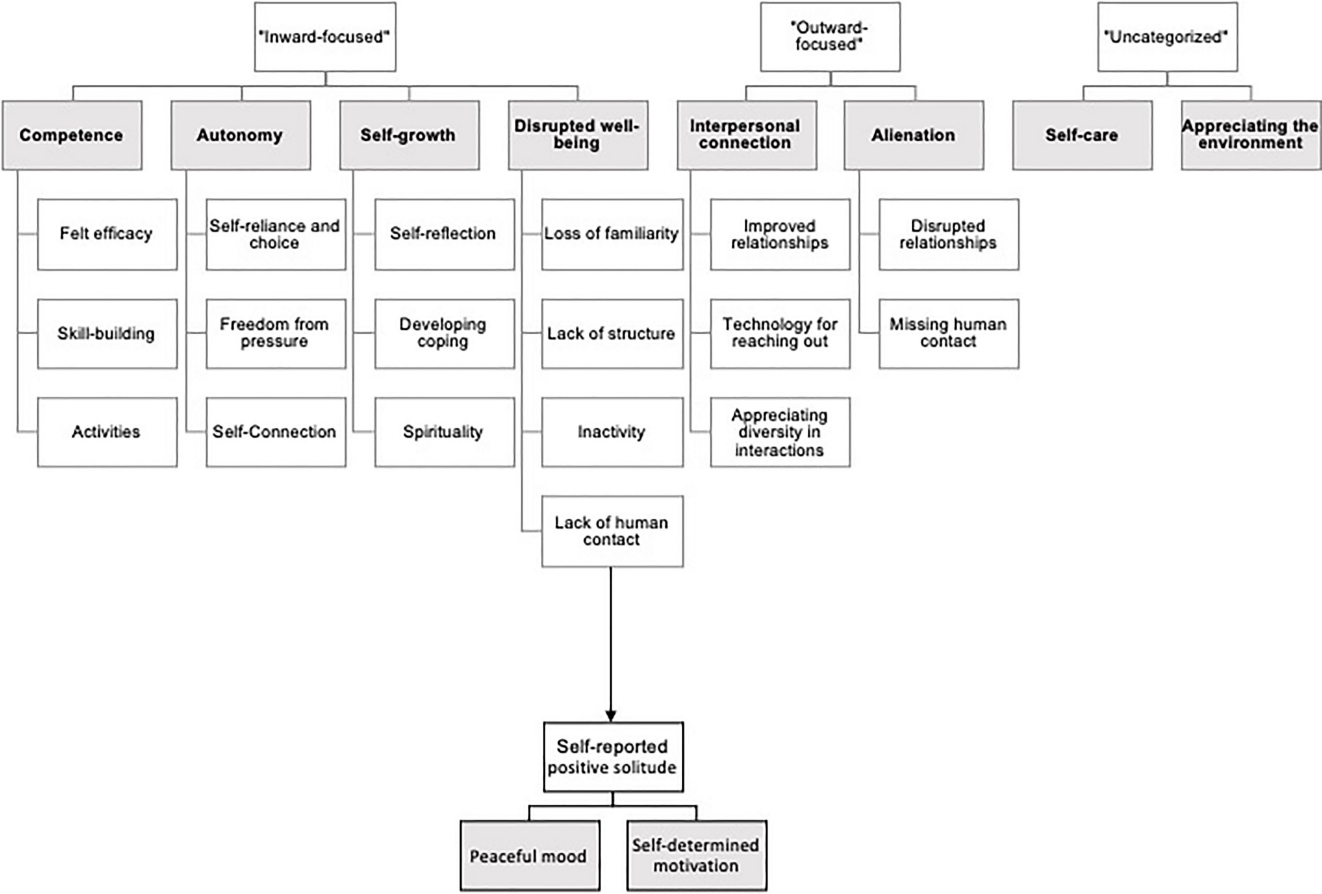
Hierarchy of codes and themes extracted from narratives through the full thematic analysis process. Grayed boxes represented overarching themes, or self-reports, which were tested as separate variables in qualitative and quantitative models.

The final themes resulting from the full thematic coding process are shown in Figure 1 in shaded gray boxes, with white boxes representing lower order themes embedded in each because doing so more accurately represented expert rating or statistical covariation.

#### Quantitative Analyses

The final set of themes and self-reports of motivation and peaceful mood were then analyzed quantitatively using SPSS-27 through several steps. First, multiple analyses of variance (MANOVA) analyses predicted themes and mood from age groups (adolescents vs. adults vs. older adults). MANOVAs first tested statistically significant omnibus effects across all outcomes (themes or self-reports) to reduce the chance of spurious effects of age. These analyses were used to describe differences in salience of themes across the different age groups (i.e., whether individuals in certain age groups would find particular consequences of their solitude to be evident and therefore mention them at higher rates).

Second, linear regression analyses were conducted to examine the practical significance of themes on well-being by testing the relations between themes and well-being in solitude measures. It was presumed that those frequent themes that relate to well-being are not only salient, but also consequential for wellness. Simultaneously, moderations by age were estimated through testing interaction terms for each theme in the same models. This additional step asked the question: does the significance of certain themes differ across age groups, such that a theme relates more strongly to self-reported well-being at certain ages? To reduce spurious effects and the number of analyses, themes were modeled simultaneously in multiple regression models.

## Results

### Final Themes

#### Inward-Focused

##### Competence

Competence involved felt efficacy, a focus on skill-building, and engagement in activities. When individuals responded that they engaged in activities and created structure in their days, they also felt they could build skills and feel effective in daily activities.

“Doing my own hobbies that I am interested in that don't require the presence of others” (female, age 15)

“I have spent my time with cooking, painting art and craft and good games videos” (male, age 15)

“I've learned some new languages and studied some new things on the internet I've been thinking after lockdown to enjoy life more” (male, age 14)

“when I'm alone I tend to learn new things, exercise more and play more music” (male, age 30)

“It's absolutely fine to be on your own, you have got more time to do things you have not got time to do, like reading or online learning” (female, age 31)

“I've learned that I prefer more productive and studious pastimes as opposed to things like watching TV and playing video games. I really only discovered this by accident; I decided to try and learn Spanish and found myself enjoying the experience” (female, age 42)

“I have caught up with a lot of reading and completed an online course in a subject that really interests me. I did well on the course and this has encouraged me to pursue the subject further” (female, age 69)

“Good - pottering in garden, being creative, listening to music, walking in the park - saying hello to people, at the end of the day having crossed most items off my to-do list, rooting out the stuff lurking at the back of my food cupboard and using it, experimenting with recipes” (female, age 62)

##### Autonomy

Themes of autonomy involved a sense of self-reliance, elimination of pressure, and self-connection. Solitude that had autonomy facilitated a connection with the self that was conducive to a sense of choice, and freedom from the pressures and responsibilities set by others.

“I like myself and my own company” (female, age 13)

“I can rely on my parents but I would prefer to do things myself and my own way” (male, age 13)

“Be more independent and think for myself” (male, age 15)

“How to love and be comfortable with my own company. It has made me more confident about myself” (female, age 30)

“I learnt to listen to my own desires, needs and wishes. I tried to enjoy the moments and the activities I really like” (male, age 36)

“Freedom to relax and be myself whilst alone. To do things that I want to do without distraction or interruption” (male, age 41)

“It has been easy as I said before, I am really comfortable with myself. [self-growth: I have the time I need to reflect on things], with no pressure. I feel in control” (female, age 44)

“I found the times on my own to be good as I was able to devote time to doing the things I enjoy” (female, age 63)

“I have been able to feel quite relaxed, feeling I had nobody to answer to but myself” (male, age 77)

##### Self-Growth

Experts agreed that self-growth was further made up of self-reflection, development of coping ability, and spirituality. Examples of these include as:

“Time spent alone has been really good to reflect on where I am going in my life” (female, age 16)

“Gives me time to work things out and collect my thoughts” (male, age 16)

“When I am alone I can reflect and organise my thoughts on my life direction and problem-solve any issues I may have been facing. I can also check-in with my plans for the future and make sure I am on the right path” (female, age 38)

“Good - acceptance and appreciation of the present moment. Presence. Meditating” (male, age 42)

“The times spent alone have allowed me to relax away from the constant demands on my time. But they've given me time to reflect on how my life has improved now that I'm married with children” (male, age 60)

“I have learned that it is nice to have some 'alone' time. It gives me time to think clearer without someone else being around interrupting my thought processes” (female, 61)

“It has been good. It has brought me a much calmer outlook to things happening in my life” (female, age 69)

##### Disrupted Well-Being

Disrupted well-being was made up the loss of familiarity, lack of structure, and inactivity, or missing human contact.

“Being alone has been bad because I sleep lots and do not do much” (male, age 13)

“It was hard when I had no structure to work towards” (female, age 14)

“My times on my own weren't too good, because I'm far from home and I literally feel like there no one that I know better in miles” (male, age 30)

“Being in solitude can have negative effect on one’s psychological or mental state thus it’s not particularly all cool to feel alone” (male, age 30)

“There have been occasions when I have been on my own with no plans and I have not left my bed all day. This day was spent procrastinating until I felt very negative about my life overall” (female, age 31)

“I have learned that on days when I am alone, if I don't plan ahead and set myself a task or reward for the day, that I will end up spending most of the day in bed, not doing anything productive. I often feel a bit down about this as I feel that I have wasted a day” (female, age 31)

#### Outward-Focused

*Interpersonal connection* involved a sense that solitude had in some way contributed to appreciating relationships, the use of technology for reaching out, and diversity in interactions.

“I have learnt I really value them and I speak to my friends online everyday as I miss them” (male, age 13)

“Connecting with friends online has made me feel good and part of a community” (female, age 15)

“Being alone from my parents has enabled me to think and reflect back upon our relationship and the parts that I value and to focus on this rather than the negative things that were missing from my childhood” (female, age 30)

“Time spend on my own is easier with video call with my family and friends” (female, 31)

“I have learned that I need people around me throughout the day. I miss the day to day conversations with people” (male, age 44)

“Being alone gives an added freedom but also brings a closer relationship to family through more regular contact” (male, age 60)

“I’ve learned that I need people more than I thought” (female, age 69)

*Alienation* was made up of disrupted relationships as a function of solitude (not coding disruptions from COVID-19, outside the context of solitude).

“I miss my friends and being able to interact with them” (male, age 14)

“I feel I have no friends” (male, age 16)

“For a long while, I was fine spending time by myself, as I am a loner by nature. However, recently I have felt very frustrated and angry that I don't have any friends to call on” (male, age 37)

“I have learned that I don't have as many good friends as I had hoped.” (female, age 44)

“… I spend a lot of time dealing with my family's needs” (female, age 60)

#### Other/Uncategorized

##### Self-Care

Self-care involved one original theme described by adults and older adults, which concerned activities focused on caring for oneself and rejuvenation.

“I focused on myself and I tried to exercise ….” (female, age 26)

“I learned to take care about my own health. To cook healthy food for myself, to exercise” (male, age 30)

“I have learned that given sufficient time to myself free of interruptions, I can be productive and get things done. I've also learned that rest is really important and that doing nothing is okay. I've learned this simply by having free time to myself and either being able to plan and get on with the mountain of jobs I have built up, or by sitting and watching Netflix when the weather is too bad to go outside” (female, age 32)

“I do things I enjoy when on my own like yoga and Pilates and this makes me find being on my own easy and enjoyable” (female, age 60)

“I’ve been attending an 8-week course *via* zoom for mindfulness and this has helped me relax and it’s my choice to be quiet and alone after my mindfulness classes. It’s good just to switch off from all that’s going on in the world” (female, age 67)

##### Appreciating the Environment

Appreciating the environment involved one original theme described by adults and older adults, which concerned activities focused on caring for oneself and rejuvenation.

“Focusing on something, for example gardening, has been an enjoyable hobby to spend this time alone. I think the act of doing something manually, and especially in contact with nature, helps grounding yourself and it sort of becomes a moment of meditation almost, when you can just free your mind from everything and just focus on the task you are doing at that moment” (female, age 26)

“I also enjoy going for quiet walks with nothing, but birds chirping and few cars” (male, age 32)

“Being alone makes you appreciate the silence and nature and it gives peace” (Female, age 36)

“I prefer to walk in the hills on long day hikes on my own, being at one with nature and feeling the bones of the land” (male, age 60)

“Good things of being alone is you can completely switch off from distractions and appreciate nature” (female, age 63)

“I have been alone when I have taken walks out in the countryside and have reflected on what is important to me” (female, age 66).

#### Frequencies of Narratives as a Function of Age

The first test involved comparing frequency of codes across age categories to test whether certain ages would recall certain benefits more frequently than others. Examining omnibus effects, themes were expressed at different rates in the different age groups, *Wilks’ Lambda F*(14, 4,058)=35.22, *p*<0.001, *d*=0.19. Results of the MANOVA also showed main effects of age group on frequencies of narrative codes (presented bottom half of Table 1), and each was further investigated in pairwise comparisons (presented top half of Table 1).

**Table 1:** Frequencies of narrative codes for each of the three age groups and their differences across age groups.

	Competence	Autonomy	Growth	Disrupted WB	Connection	Alienation	Self-care^	Appreciat. Environ^	Negative Mood
Adolescents	42.1_A_	22.8_A_	37.6_A_	29.4_A_	39.4_B_	14.7_C_	–	–	27.8_A_
Adults	48.9_B_	54.7_B_	61.6_B_	35.6_B_	36.7_B_	7.1_B_	18.9_B_	5.7_A_	44._B_
Older adults	41.9_A_	53.8 _B_	43.2_A_	23.7_A_	20.0_A_	2.3_A_	6.8_A_	6.8_A_	24.5_A_
**Full sample^**	**43.8%**	**38.8%**	**45.2%**	**29.5%**	**33.9%**	**9.7%**	**6.6%**	**3.2%**	**30.8%**
*F Age*	3.78	118.31	42.82	8.85	30.84	33.51	34.48	0.54	30.00
*p Age*	0.023	<0.001	<0.001	<0.001	<0.001	<0.001	<0.001	0.461	<0.001
*Cohen’s d* ^*^	0.09	0.48	0.29	0.13	0.24	0.25	0.36	0.04	0.24

*F*, *p*, and *d* were determined from MANOVA analyses predicting each theme from the three age groups. ^Full sample is the percent across the sample (note, adolescents are disproportionately represented). _ABC_ letters denote statistical significance when different (e.g., _A_ vs. _B_) and no differences when the same. Letters down the alphabet (_A, B, C_) represent higher percentages. ^These codes were also identified in adults and older adults and were conducted in a separate MANOVA because they were only available for adults and older adults.

* Cohen’s *d* refers to the omnibus effect size across all ages. Bolded figures are for the full sample across adolescents, adults, and older adults.

From pairwise comparisons, a number of themes were described most often by adults, when compared to adolescents or older adults. Notably, adults described *competence* themes at significantly higher rates (48.9%) than the other groups, although competence was seen as a common benefit of solitude in other age groups as well (41.9% of older adults and 42.1% of adolescents). Adults also described *self-growth* as a benefit of solitude at higher rates (61.6%) than either of the other age groups (37.6–43.2%), and they described more *self-care* as a frequent benefit (18.9% of adults described solitude as a place for self-care). However, adults also described more *disrupted well-being* (adults: 35.6%; vs. 23.7 and 29.4%), suggesting that alongside the beneficial themes reviewed above, they also thought more about the costs of solitude.

Notably, where adolescents differed was on the salience of *autonomy* themes. Across adulthood, autonomy as a benefit of solitude was a common theme (53.8–54.7%), which was apparent, but less frequent, in adolescents (22.8%).

Finally, older adults described fewer relational costs and benefits. Specifically, older adults placed the least emphasis on solitude as making a greater contribution to relationships (i.e., *interpersonal connection*, 20.0%) and also were less likely to describe *alienation* as a theme of solitude (2.4%). In summary, older adults focused heavily on *autonomy* themes and were least likely to discuss relationship themes (either in a positive or negative light).

#### Self-Reported Well-Being in Solitude as a Function of Age

Table 2 summarizes patterns for the two self-reported indicators of well-being in solitude which differed across the three age groups (Table 2). Adolescents reported the least *self-determined motivation*, followed by older adults. However, although adults reported the highest levels of *self-determined motivation* as compared to the other two age groups, they also reported the least *peaceful mood*. Despite concerns about solitude in older adulthood (e.g., Rokach, 2015), older adults reported the most relaxed, least lonely mood, when alone.

**Table 2:** Self-reported well-being for each of the three age groups.

	Motivation *M* (*SD*)	Peaceful Mood *M* (*SD*)
Adolescents	4.90_A_ (1.16)	4.89_A_ (1.17)
Adults	5.47_C_ (1.25)	4.74_A_ (1.38)
Older adults	5.07_B_ (1.56)	5.33_B_ (1.44)
*F*	32.82	28.86
*p*	<0.001	<0.001
*d* ^*^	0.25	0.24

Motivation=self-determined motivation. _ABC_ letters denote statistical significance when different (e.g., _A_ vs. _B_) and no differences when the same. Letters down the alphabet (_A, B, C_) represent progressively higher means.

* Cohen’s *d* refers to the omnibus effect size across all ages.

#### Primary Quantitative Models

**Figure 2:**
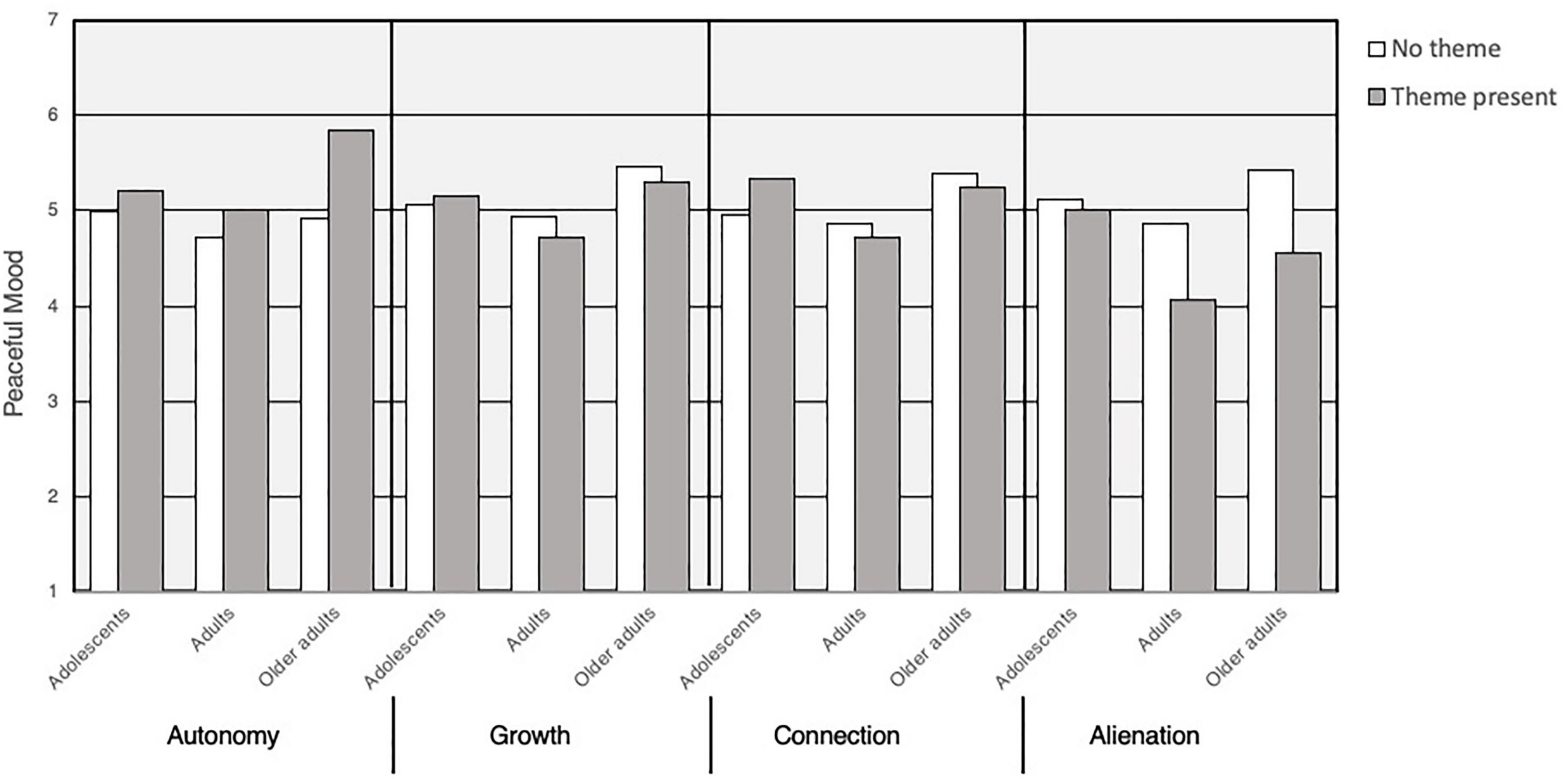
Interaction effects: means of peaceful mood (y axis) as a function of each theme [absent (white) or present (gray)], and separately adolescents, adults, and older adults (x axis). Means were derived from PROCESS moderation analyses for illustration purposes and may slightly differ from simultaneous regression models.

Hierarchical linear regression models predicted the three self-report composites concerning motivation and mood in solitude from coded narratives. Step 1 included main effect predictors across the lifespan, and Step 2 included age groups as a separate dummy-coded predictor. In line with the pre-registered analytic plan set before the start of the study: Dummy code 1 contrasting adolescents to adults: adolescents (*1*) vs. adults (*0*) and older adults (*0*): Dummy code 2 contrasting older adults to adults: older adults (*1*) vs. adults (*0*) and younger adults (*0*). In a final set of regression models, interaction effects of age group X narrative code were tested predicting the self-reported well-being.^8^ Summarizing these findings, Table 3 presents main effects across all age groups, Table 4 presents interaction effects by age group, and Table 5 presents the simple effects within each age group. To illustrate these interactions, Figure 2 also presents the means of peaceful mood as a function of age and theme (present or absent) for four themes: autonomy, growth, connection, and alienation.

**Table 3:** Main effects predicting well-being in solitude indicators: peaceful mood and self-determination motivation.

	Main Effect					
	*β*	*b*	*Se-b*	*t*	*p*	*pr*
**Peaceful Mood**						
Competence	0.02	0.06	0.06	1.12	0.265	0.02
**Autonomy**	**0.17**	0.47	0.06	**7.82**	**<0.001**	0.17
**Growth**	**−0.05**	−0.12	0.06	**−2.11**	**0.035**	**−0.05**
Connection	0.03	0.09	0.06	1.46	0.145	0.03
**Alienation**	**−0.07**	−0.31	0.10	**−3.20**	**0.001**	**−0.07**
**Disrupted WB**	**−0.17**	−0.48	0.06	**−7.67**	**<0.001**	**−0.17**
Self-care	0.03	0.13	0.13	1.06	0.289	0.03
App. Environ	0.01	0.04	0.18	0.24	0.812	0.01
**Self-Determined Motivation**						
Competence	0.02	0.06	0.06	1.06	0.289	0.02
**Autonomy**	**0.26**	0.70	0.06	**11.90**	**<0.001**	**0.25**
**Growth**	**0.05**	0.12	0.06	**2.09**	**0.036**	**0.04**
**Connection**	**0.06**	0.18	0.06	**2.99**	**0.003**	**0.06**
Alienation	−0.03	−0.14	0.10	−1.49	0.136	−0.03
**Disrupted WB**	**−0.13**	−0.38	0.06	**−6.16**	**<0.001**	**−0.13**
**Self-care**	**0.12**	0.51	0.12	**4.14**	**<0.001**	**0.12**
App. Environ.	0. 03	0.17	0.17	0.97	0.331	0.03

Statistically significant main effects are bolded. Disrupted WB=Disrupted well-being. App. Environ.=appreciating the environment.

**Table 4:** Interaction effects predicting self-reports of solitude from coded narrative themes. Motivation and mood are defined as outcomes in the model.

Vs. adults	Peaceful Mood						Self-Determined Motivation					
	Adolescents			Older adults			Adolescents			Older adults		
	*β*	*t*	*p*	*β*	*t*	*p*	*β*	*t*	*p*	*β*	*t*	*p*
Competence	0.00	−0.05	0.962	0.02	0.45	0.655	−0.03	−0.82	0.412	−0.06	−1.52	0.129
Autonomy	−0.03	−0.91	0.363	**0.13**	**3.1**	**0.002**	−0.05	−1.51	0.131	**0.23**	**5.60**	**<0.001**
Growth	**0.11**	**2.56**	**0.01**	0.03	0.79	0.427	**0.09**	**2.08**	**0.038**	0.03	0.69	0.491
Connection	**0.15**	**3.43**	**0.001**	0.02	0.65	0.515	**0.08**	**1.80**	**0.071**	0.02	0.71	0.479
Alienation	**0.12**	**2.51**	**0.012**	0.00	0.19	0.847	**0.12**	**2.53**	**0.011**	0.01	0.33	0.744
Disrupted WB	−0.01	−0.24	0.81	**−0.09**	**−2.97**	**0.003**	−0.07	−1.79	0.074	−0.09	−3.03	0.002
Self-care				0.03	1.06	0.289				**0.07**	**2.06**	**0.040**
App. Environ.				0.01	0.24	0.812				**0.06**	**1.42**	**0.157**

Narratives codes (left panel) were defined as simultaneous predictors in the model, and dummy codes were used to compare adults to adolescents and older adults. Statistically significant interaction effects are bolded. Disrupted WB=Disrupted well-being. App. Environ.=appreciating the environment.

**Table 5:** Simple slopes contrasting each pair of age groups (adults vs. adolescents and adults vs. older adults) predicting well-being in solitude: peaceful mood and self-determination motivation.

	Interactions of adults vs. (as *detailed in* Table 4)		Simple slopes (*β*)		
Adoles	Older adults	Adoles	Adult	Older
**Peaceful Mood**					
Competence	ns	ns	0.03	0.03	0.05
Autonomy	ns	*p*<0.05	**0.08**	**0.13**	**0.29**
Growth	*p*<0.05	ns	0.04	**−0.10**	−0.04
Connection	*p*<0.05	ns	**0.14**	−0.05	−0.01
Alienation	*p*<0.05	ns	−0.04	**−0.14**	−0.08
Disrupted WB	ns	*p*<0.05	**−0.13**	**−0.11**	**−0.24**
Self-care	NA	ns		0.04	**0.10**
App. Environ.	NA	ns		−0.01	−0.02
**Self-Determined Motivation**					
Competence	ns	ns	0.03	0.08	−0.04
Autonomy	ns	*p*<0.05	**0.08**	**0.17**	**0.41**
Growth	*p*<0.05	ns	**0.09**	−0.05	0.02
Connection	*p*<0.05	ns	**0.10**	0.00	0.04
Alienation	*p*<0.05	ns	0.01	**−0.12**	−0.05
Disrupted WB	ns	*p*<0.05	**−0.16**	−0.06	**−0.18**
Self-care	NA	*p*<0.05		0.08	**0.13**
App. Environ	NA	ns		−0.01	0.07

Adoles=adolescents; older=older adults. Interaction effects from Table 4 are summarized on the left-hand columns, separately comparing adults to adolescents and adults to older adults. ns=not significant at *p*<0.05. NA=the interaction could not be tested because themes were not identified in adolescents. Bolded simple slopes represent statistically significant simple slopes at *p*<0.05. Disrupted WB=disrupted well-being. App. Environ.=appreciating the environment.

Across all age groups (when examining main effects across the lifespan; Table 3), the most robust relation across the lifespan involved autonomy themes. Those who described autonomy as a benefit of solitude also reported more peaceful mood during solitude (*β*=0.17) and more self-determined motivation for solitude (*β*=0.26).

Examining moderation effects, the links between autonomy narratives and self-reported well-being in solitude interacted with age in the “older adult” versus “adult” comparison only (interaction effects in Table 4; simple effects in Table 5). In other words, interaction effects showed that autonomy themes were more strongly correlated with well-being for older adults as compared to adults across both self-reported well-being measures; The difference was not statistically significant when comparing adolescents to adults.

A second significant pattern across the lifespan was observed for the narrative code “disrupted well-being,” which involved loss of routine, missing the familiar, missing human contact, and inactivity. Across the lifespan, disrupted well-being was related to lower well-being (Table 3): less peaceful mood during solitude (*β*=−0.17) and less self-determined motivation for solitude (*β*=−0.13). The links between disrupted well-being narratives and more detrimental self-reported outcomes in solitude were stronger for older adults than adults (interaction effects in Table 4; simple effects Table 5).

Relationships with others were reflected in seeking positive interpersonal connection and, also, felt alienation. *Alienation* correlated with less peaceful mood across the lifespan (*β*=−0.07), but the strongest negative relations between alienation and mood were found for adults. Across the lifespan, *connection* correlated with more self-determined motivation (*β*=0.06). Further examining simple slopes, adolescents who described connection reported consistent and robust wellness benefits during solitude. Only adolescents benefited when discussing connection themes, and they drove the main effect observed across the lifespan.

Growth, involving self-reflection, developing coping, and spirituality, showed intriguing inconsistencies. Across the lifespan, growth correlated with more self-determined motivation (*β*=0.05), yet it was linked to less peaceful mood during solitude (*β*=−0.05). The adolescent versus adult code moderated the relation between growth themes and self-reported well-being. Examining this further, adolescents showed a significant *positive* link between growth themes and self-determined motivation, and a positive trend between growth and peaceful mood. On the other hand, adults reported *negative* trends on both measures.

Finally, no main effects were observed for self-care, appreciating the environment, or competence, although in older adults self-care was related to more self-reported well-being in solitude. For the first two of these constructs, this may be due to the very low rates (around 5–7%) of these narratives with that code. The lack of relations with competence was surprising, because it was reported at relatively high rates (around 40%) across the lifespan. Thus, it was a commonly reported benefit of solitude, but not one that translated to more self-reported well-being.

## Discussion

This mixed-method research was designed to define the salience (qualitatively) and significance (quantitatively) of benefits and costs of solitude in order to build a richer understanding of how solitude is experienced across the lifespan. Over 2,000 individuals in their adolescence, adulthood, or older adulthood described the gains, losses, and lessons learned from solitude. To define the salience of specific experiences in solitude, the frequencies by which themes were reported in the narratives of individuals from these different age groups were tested, as were convergences between extracted themes and self-reported well-being (self-determined motivation and peaceful mood) in solitude.

Differences among age groups were evident in survey responses. Adults reported the most self-determined motivation for solitude, but the least peaceful mood. Older adults reported the least self-determined motivation for solitude when compared to adults, but the most peaceful mood when alone. This finding stands in contrast with qualitative work showing older adults are likely to choose solitude (Ost Mor et al., 2020), but the difference likely lies in the timing of this study, the COVID-19 outbreak, which limited choice and opportunities for many older adults who were self-shielding (Robb et al., 2020). It is noteworthy that despite their lower self-determined motivation, older adults still reported their time alone as more peaceful than adults or adolescents. The latter finding aligns well with a body of work suggesting older adults are better at enjoying time alone (Larson, 1990; Lay et al., 2019; Ost Mor et al., 2020). Solitude is often viewed as a positive state (Long et al., 2003; Ost Mor et al., 2020), and it is also noteworthy that consistent with this view all three age groups – adolescents to older adults – generally reported above-mean (at approximately 5 on a 7-point scale) levels of self-determined motivation and peaceful mood. Though instructions carefully avoided biasing participants toward expressing solitude as a positive phenomenon in their lives, on the whole, most expressed that it is. In sum, solitude was, on the whole, reported to be a positive experience across the lifespan but was most peaceful for older adults, despite reduced self-determined motivation likely due to COVID-19 restricting social interactions.

Through the process of coding narratives, overarching themes were identified that characterized gains, losses, and lessons learned. Three prominent themes extracted were consistent with views of self-determination theory (Ryan and Deci, 2000a,b), a guiding framework for the lead researcher but one to which coders was intentionally naïve. SDT posits that three basic psychological needs shape well-being and flourishing: competence – feeling effective in the things one does; autonomy – feeling that one is volitional and choiceful in one’s actions and congruent with oneself; and relatedness – experiencing a sense of closeness and connection with others (Deci and Ryan, 2008b; Church et al., 2013; Milyavskaya et al., 2013). To the extent that those needs are met, individuals experience higher well-being; and to the extent they are thwarted, individuals’ well-being is undermined (Ryan and Deci, 2017).

Although psychological need satisfaction has not been studied in the context of solitude, it is reasonable to assume that one could feel more or less psychologically need-satisfied when alone. For example, when alone one may feel free to be oneself and undertake tasks deemed interesting or important. This autonomy satisfaction *in* solitude can be distinguished from self-determined motivation *for* solitude, which concerns one’s choice to volitionally enter into solitude; arguably, both are important (Weinstein et al., in press).

Previous research (Ammaniti et al., 1988) and theorizing (Larson, 1997) suggest that solitude is important for experiencing autonomy in adolescence. The SDT literature also posits that feeling choiceful and free from pressure is important for well-being across the lifespan (Ryan et al., 1995). In the current study, autonomy was identified to be an affordance of solitude by around 20% of adolescents but was more frequently described by around 50% of adults and older adults. Thus, across the adult lifespan, autonomy was seen as a salient affordance of time spent alone. Consistent with previous theorizing (e.g., Ryan et al., 1995), autonomy was also a significant predictor of well-being. Those who described autonomy as an affordance, regardless of their age, also reported higher well-being in solitude, though the relation between autonomy themes and self-reported well-being in solitude was stronger in older adults than it was for adolescents.

While this was the case in the current study, autonomy seemed to play a particularly important and beneficial role for older adults that shaped how they felt when alone and was partly responsible for a “peaceful mood” during their solitude. One explanation for increasing associations between the autonomy theme and well-being across the lifespan is that, since self-connection and self-initiation are still naturally developing at different rates in young people from age 13 to 20 years (Collins et al., 1997), the successful resolution of this process may play a more central role in determining well-being in solitude for adults.

Alongside experiencing autonomy, even when individuals are alone, they may still seek and value connection to others since relatedness is a fundamental human need (Baumeister and Leary, 1995; Ryan and Deci, 2000a), and because interpersonal connections are carried through memories and residual impressions of social experiences (Weinstein, 2014). In this work, individuals describing solitude expressed a desire to connect with others. Narratives discussed seeking connections, or feeling alienated, from others. The theme of alienation speaks to the extant literature that views solitude in terms of social withdrawal or otherwise disrupted relationships (Rubin et al., 2014). However, it is worth bearing in mind that during the pandemic, participants may have been experiencing non-typical solitude that disrupted the balance between social and alone time (Robb et al., 2020). Also noteworthy, themes of connection and alienation differed across the lifespan. Of the three age groups, older adults were least likely to describe desire for connection or alienation themes. Therefore, for older adults, solitude was generally most divorced from social interactions, a view which is aligned with previous research showing that older adults are less reliant on others’ presence (Carstensen, 1992; Palmonari, 2011; Lay et al., 2019).

Although adolescents did not differ from adults in terms of the salience of connecting to others when alone (both reported interpersonal connection at relatively high rates), they were more likely than adults to spontaneously discuss feeling alienated in solitude. It may be that adolescents are finding time alone particularly disruptive to their relationships, a developmentally appropriate reaction given that a primary task of adolescent development is constructing and understanding themselves in their friendships (Hartup, 1989). Further, adolescents benefited, reporting more self-determined motivation and peaceful mood, when they perceived connections to others.

The final of the three psychological needs, competence, can be readily experienced in solitude when individuals undertake activities and tasks that are challenging but achievable (Ryan and Deci, 2000a). Indeed, competence was identified with some frequency (app. 40% of respondents) across age groups. SDT argues that competence is an important precursor of well-being (Deci and Ryan, 2008b); however, there was no evidence linking competence themes with well-being at any age group. This divergence from expectation may be due to the source of competence themes; namely, some described school or work activities during COVID-19 lockdowns that were perhaps better completed with others. Thus, task-focused activities and even efficacy perceived during those activities may not necessarily have been experienced as satisfying the need for competence in the same way that self-directed competence activities are experienced (Baard et al., 2004).

Adults appeared to describe the most intrapersonal movement and upheaval during their solitude time. Compared to both adolescents and older adults, adults described more frequent growth themes: They engaged in more self-reflection, and they developed more patience and engaged spirituality. At the same time, adults also reported more disrupted well-being: They felt aimless and without structure, and they described the sense that familiarity was disrupted by their time spent alone. While growth has the potential for long-term benefits through learning about the self, emotions, and goals (Ardelt and Grunwald, 2018), it often comes at a cost for well-being in the short-term (Weinstein and Hodgins, 2009). For example, thinking about oneself can bring up painful thoughts that must be processed or may be ruminative, with mixed or no relations with well-being (Harrington and Loffredo, 2010). In line with this view, adults who described growth in solitude had lower self-determined motivation to be alone.

Finally, although self-care and appreciating the environment were two themes that emerged for adults and older adults, they were not as highly represented as one might expect. This may be because the study questions prompted reflection of what is gained and learned from solitude, rather than delving into the context that gives rise to positive solitude, where, for example, appreciating the environment may be more salient. Specifically, when individuals have nature around them, they report their time to be more peaceful and rewarding (Borrie and Roggenbuck, 2001; Long and Averill, 2003). Similarly, self-compassion and self-care are important indicators of well-being outside the context of solitude (Zessin et al., 2015). Arguably, neither of these experiences may be unique to solitude, and future research may explore the role of both self-care and the environment in social relationships and when alone.

### Limitations

These set of findings should be viewed in light of several limitations of the research. First, data were collected over a single time point and participants were asked to reflect back to solitude over a period of time. While this narrative approach is not particularly problematic in light of the current focus on identifying the most salient recollections of solitude, this research would be well suited to ecological momentary assessments (daily diary studies) that ask participants to reflect back to recent solitude and describe it soon after the alone event took place. Such an approach would pick up on more nuanced benefits and costs that may be forgotten over the course of time. It would allow for more time-specific correlations between themes and affect as a function of a specific occurrence of solitude rather than across time, correlations which will likely be stronger as they capture a specific and well-defined experience.

Second, though the study used a mixed-methods approach that reduced common method variance (Podsakoff et al., 2003), the correlational nature of the data precluded casual interpretations regarding themes that emerged. As such, future research should consider experimental interventions to change participants’ framing for solitude in light of the themes that emerged here. For example, Nguyen et al. (2021) have done similar work manipulating motivational framing of solitude in terms of self-determined or non-self-determined motivation. It may be that making certain affordances of solitude salient – such as autonomy across the lifespan, or growth in a sample of adolescents – could serve as an experimental manipulation that might shape consequent well-being in solitude. This approach would help to address potential alternative explanations for the effects identified in this study.

Further, this research involved young- to mid- adolescents aged 13–16 years, but evidence suggests that the capacity to enjoy solitude develops throughout adolescence, and older adolescents are more like to benefit from time spent alone (Larson, 1990; Coplan et al., 2019). Older adolescents – a sample not tested in this study – may have demonstrated benefits similar to those reported by the adult sample. Finally, because this study was conducted during the early months of the COVID-19 outbreak, it is unclear the extent to which results would generalize beyond this period to everyday solitude. However, the benefits described by participants *despite* lockdowns during this time are particularly informative for understanding the potential gains from time spent alone.

### Conclusion

This study highlighted potential benefits, as well as costs, of solitude and how they vary over three age groups. Alongside understanding findings in light of self-determination theory, the study findings converged with the affordances described by Long et al.’s (2003) study with college students. Specifically, Long’s distinction between inward-focused affordances (e.g., autonomy) and outward-focused affordances (e.g., connection) continued to be a useful framework aligned with these findings. Furthermore, many of the themes Long identified were evident in the current research: “solitude as anonymity” – acting without concern for social niceties – mapped closely onto the theme of “freedom from pressure”; “solitude as intimacy” corresponded with “connection”; “solitude as creativity” corresponded with some aspects of the competence theme; and “solitude as problem-solving and self-discovery” mapped well onto the personal growth themes. Diverging from Long et al. (2003), who described all affordances as benefiting college students’ well-being, they were observed at different rates and correlated differently with well-being across the lifespan, and as a function of age groups.

In short, adolescents (age 13–16 years) had little interest in the *autonomy* offered by solitude but were interested in its opportunities to feel *competent* and to experience *self-growth*. They were also less likely to feel *disrupted well-being* in solitude, and more likely to experience a feeling of *interpersonal connections* during that time. Adults (age 25–51) were invested in solitude primarily as a place of *self-growth* and *competence* where they could focus on skill-building and feel effective and engaged. Unlike adolescents, but similar to their elders, adults were particularly invested in reaping the benefits of *autonomy* in solitude. Many felt connected to others but are also the most likely of the three groups to experience *disrupted well-being* in solitude. Finally, older adults (age 59–85) were slightly less interested in *competence* and much less interested in *self-growth* possible in solitude. But they were invested in *autonomy* – a space in which they could feel self-reliant and connect with themselves, free from the pressure of others.

## Data Availability Statement

The raw data supporting the conclusions of this article will be made available by the authors, without undue reservation.

## Ethics Statement

The studies involving human participants were reviewed and approved by the University of Reading School of Psychology Ethics Committee (no 2020-080-NW). Written informed consent to participate in this study was provided by the participants’ legal guardian/next of kin.

## Author Contributions

NW, HH, and T-VN conceptualized the project and developed the methodology. NW conducted data collection, organized coders, led data analysis, and led manuscript preparation with active contributions from HH and T-VN. HH and T-VN supported data analysis and interpretation. All authors contributed to the article and approved the submitted version.

## Funding

This research was funded by the ERC grant SOAR-859810 awarded to the NW.

## Conflict of Interest

The authors declare that the research was conducted in the absence of any commercial or financial relationships that could be construed as a potential conflict of interest.

## Publisher’s Note

All claims expressed in this article are solely those of the authors and do not necessarily represent those of their affiliated organizations, or those of the publisher, the editors and the reviewers. Any product that may be evaluated in this article, or claim that may be made by its manufacturer, is not guaranteed or endorsed by the publisher.

## Footnotes

^1^Doi: 10.17605/OSF.IO/B2AMQ The only notable deviation from registered plans is that we do not discuss results with self-reported need satisfaction. Their findings were consistent with peaceful mood. However, self-reported need satisfaction was redundant with the themes that emerged from narrative coding and had an unclear position in the model.

^2^Exploratory question, as it was not registered prior to data collection.

^3^We had set an inclusion criterion for participants to be between the ages of 30–50 years, but six participants outside this (25–29 or 31 years) age signed up for the study.

^4^We had set an inclusion criterion that participants are aged 60+ years, but received one respondent of age 59.

^5^ https://www.icmunlimited.com

^6^As a note, reliability for this measure was lower in adolescents and adults as compared to adults, suggesting variance across age groups. Correlations with this measure should be interpreted with caution and alongside results for affect.

^7^Doi:10.17605/OSF.IO/B2AMQ.

^8^Self-care and appreciating the environment were tested in a separate model including all predictors but no dummy codes for adolescents.

## References

[ref1] Ammaniti M. Ercolani A. P. Tambelli R. (1988). Loneliness in the female adolescent. J. Youth Adolesc. 18, 321–329. doi: 10.1007/BF02139252, PMID: 24271917

[ref2] Ardelt M. Grunwald S. (2018). The importance of self-reflection and awareness for human development in hard times. Res. Hum. Dev. 15, 187–199. doi: 10.1080/15427609.2018.1489098

[ref3] Baard P. P. Deci E. L. Ryan R. M. (2004). Intrinsic need satisfaction: a motivational basis of performance and weil-being in two work settings 1. J. Appl. Soc. Psychol. 34, 2045–2068. doi: 10.1111/j.1559-1816.2004.tb02690.x

[ref4] Baumeister R. F. Leary M. R. (1995). The need to belong: desire for interpersonal attachments as a fundamental human motivation. Psychol. Bull. 117, 497–529. doi: 10.1037/0033-2909.117.3.497, PMID: 7777651

[ref5] Borrie W. T. Roggenbuck J. W. (2001). The dynamic, emergent, and multi-phasic nature of on-site wilderness experiences. J. Leis. Res. 33, 202–228. doi: 10.1080/00222216.2001.11949938

[ref6] Buchholz E. S. (1997). The Call of Solitude: Alonetime in a World of Attachment. New York: Simon and Schuster.

[ref7] Buchholz E. S. Catton R. (1999). Adolescents’ perceptions of aloneness and loneliness. Adolescence 34, 203–204. PMID: 10234378

[ref8] Burger J. M. (1995). Individual differences in preference for solitude. J. Res. Pers. 29, 85–108. doi: 10.1006/jrpe.1995.1005

[ref9] Cacioppo J. T. Hawkley L. C. (2009). “Loneliness,” in Handbook of Individual Differences in Social Behavior. eds. Leary M. R. Hoyle R. H. (New York: The Guilford Press), 227–240.

[ref10] Carstensen L. L. (1992). Social and emotional patterns in adulthood: support for socioemotional selectivity theory. Psychol. Aging 7, 331–338. doi: 10.1037/0882-7974.7.3.331, PMID: 1388852

[ref11] Church A. T. Katigbak M. S. Locke K. D. Zhang H. Shen J. de Jesús Vargas-Flores J.. (2013). Need satisfaction and well-being: testing self-determination theory in eight cultures. J. Cross-Cult. Psychol. 44, 507–534. doi: 10.1177/0022022112466590

[ref12] Collins W. A. Gleason T. Sesma A. Jr. (1997). “Internalization, autonomy, and relationships: development during adolescence,” in Parenting and Children's Internalization of Values: A Handbook of Contemporary Theory. eds. Grusec J. E. Kuczynski L. (USA: John Wiley & Sons Inc.), 78–99.

[ref13] Coplan R. J. Bowker J. C. (eds.) (2013). The Handbook of Solitude: Psychological Perspectives on Social Isolation, Social Withdrawal, and Being Alone. USA: John Wiley & Sons.

[ref14] Coplan R. J. Ooi L. L. Baldwin D. (2019). Does it matter when we want to be alone? Exploring developmental timing effects in the implications of unsociability. New Ideas Psychol. 53, 47–57. doi: 10.1016/j.newideapsych.2018.01.001

[ref15] Coplan R. J. Ooi L. L. Nocita G. (2015). When one is company and two is a crowd: why some children prefer solitude. Child Dev. Perspect. 9, 133–137. doi: 10.1111/cdep.12131

[ref16] Corsano P. Majorano M. Champretavy L. (2006). Psychological well-being in adolescence: the contribution of interpersonal relations and experience of being alone. Adolescence 41, 341–353. PMID: 16981621

[ref17] Creswell J. W. Plano Clark V. L. (2011). Designing and Conducting Mixed Methods Research. 2nd *Edn*. Thousand Oaks, CA: Sage.

[ref18] Deci E. L. Ryan R. M. (2008a). Self-determination theory: A macrotheory of human motivation, development, and health. Canadian. psychol. 49, 182–185. doi: 10.1037/a0012801

[ref19] Deci E. L. Ryan R. M. (2008b). Facilitating optimal motivation and psychological well-being across life’s domains. Canadian psychol. 49, 14–23. doi: 10.1037/0708-5591.49.1.14

[ref20] Galanaki E. (2004). Are children able to distinguish among the concepts of aloneness, loneliness, and solitude? Int. J. Behav. Dev. 28, 435–443. doi: 10.1080/01650250444000153

[ref21] Galanaki E. (2005). Solitude in the school: A neglected facet of Children’s development and education. Child. Educ. 81, 128–132. doi: 10.1080/00094056.2005.10522255

[ref22] Goossens L. (2018). Loneliness in adolescence. Insights from Cacioppo's evolutionary model. Child Dev. Perspect. 12, 230–234. doi: 10.1111/cdep.12291

[ref23] Graham J. W. Taylor B. J. Olchowski A. E. Cumsille P. E. (2006). Planned missing data designs in psychological research. Psychol. Methods 11, 323–343. doi: 10.1037/1082-989X.11.4.323, PMID: 17154750

[ref24] Harrington R. Loffredo D. A. (2010). Insight, rumination, and self-reflection as predictors of well-being. J. Psychol. 145, 39–57. doi: 10.1080/00223980.2010.528072 21290929

[ref25] Hartup W. W. (1989). Social relationships and their developmental significance. Am. Psychol. 44, 120–126. doi: 10.1037/0003-066X.44.2.120

[ref26] Hawkley L. C. Cacioppo J. T. (2010). Loneliness matters: A theoretical and empirical review of consequences and mechanisms. Ann. Behav. Med. 40, 218–227. doi: 10.1007/s12160-010-9210-8, PMID: 20652462 PMC3874845

[ref27] Heinrich L. M. Gullone E. (2006). The clinical significance of loneliness: A literature review. Clin. Psychol. Rev. 26, 695–718. doi: 10.1016/j.cpr.2006.04.002, PMID: 16952717

[ref28] Holgado-Tello F. P. Chacón-Moscoso S. Barbero-García I. Vila-Abad E. (2010). Polychoric versus Pearson correlations in exploratory and confirmatory factor analysis of ordinal variables. Qual. Quant. 44, 153–166. doi: 10.1007/s11135-008-9190-y

[ref29] Howe K. R. (1988). Against the quantitative-qualitative incompatibility thesis or dogmas die hard. Educ. Res. 17, 10–16. doi: 10.3102/0013189X017008010

[ref30] Johnson R. B. Onwuegbuzie A. (2004). Mixed methods research: a research paradigm whose time has come. Educ. Res. 33, 14–26. doi: 10.3102/0013189X033007014

[ref31] Larson R. W. (1990). The solitary side of life: An examination of the time people spend alone from childhood to old age. Dev. Rev. 10, 155–183. doi: 10.1016/0273-2297(90)90008-R

[ref32] Larson R. W. (1997). The emergence of solitude as a constructive domain of experience in early adolescence. Child Dev. 68, 80–93. doi: 10.2307/1131927, PMID: 9084127

[ref33] Lay J. C. Pauly T. Graf P. Biesanz J. C. Hoppmann C. A. (2019). By myself and liking it? Predictors of distinct types of solitude experiences in daily life. J. Pers. 87, 633–647. doi: 10.1111/jopy.12421 30003553

[ref34] Lay J. C. Pauly T. Graf P. Mahmood A. Hoppmann C. A. (2020). Choosing solitude: age differences in situational and affective correlates of solitude-seeking in midlife and older adulthood. J. Gerontol. B Psychol. Sci. Soc. Sci. 75, 483–493. doi: 10.1093/geronb/gby044 29669095

[ref35] Long C. R. Averill J. R. (2003). Solitude: An exploration of benefits of being alone. J. Theory Soc. Behav. 33, 21–44. doi: 10.1111/1468-5914.00204

[ref36] Long C. R. Seburn M. Averill J. R. More T. A. (2003). Solitude experiences: varieties, settings, and individual differences. Personal. Soc. Psychol. Bull. 29, 578–583. doi: 10.1177/0146167203029005003, PMID: 15272992

[ref37] Marcoen A. Goossens L. Caes P. (1987). Loneliness in pre-through late adolescence: exploring the contributions of a multidimensional approach. J. Youth Adolesc. 16, 561–577. doi: 10.1007/BF02138821, PMID: 24277491

[ref38] Matias G. P. Nicolson N. A. Freire T. (2011). Solitude and cortisol: associations with state and trait affect in daily life. Biol. Psychol. 86, 314–319. doi: 10.1016/j.biopsycho.2010.12.011, PMID: 21262315

[ref40] Milyavskaya M. Philippe F. L. Koestner R. (2013). Psychological need satisfaction across levels of experience: their organization and contribution to general well-being. J. Res. Pers. 47, 41–51. doi: 10.1016/j.jrp.2012.10.013

[ref42] Nguyen T. V. T. Ryan R. M. Deci E. L. (2018). Solitude as an approach to affective self-regulation. Personal. Soc. Psychol. Bull. 44, 92–106. doi: 10.1177/0146167217733073 29072531

[ref43] Nguyen T. V. T. Weinstein N. Ryan R. M. (2021). “The possibilities of aloneness and solitude: developing an understanding framed through the lens of human motivation and needs,” in The Handbook of Solitude: Psychological Perspectives on Social Isolation, Social Withdrawal, and Being Alone. eds. Coplan R. J. Bowker J. C. Nelson L. J. (USA: John Wiley & Sons, Inc.), 224–239.

[ref44] Onwuegbuzie A. J. Johnson R. B. (2006). The validity issue in mixed research. Res. Sch. 13, 48–63.

[ref45] Ost Mor S. Palgi Y. Segel-Karpas D. (2020). The definition and categories of positive solitude: older and younger adults’ perspectives on spending time by themselves. Int. J. Aging Hum. Dev.:91415020957379. doi: 10.1177/0091415020957379, [Epub ahead of print] 32938200

[ref46] O’Súilleabháin P. S. Gallagher S. Steptoe A. (2019). Loneliness, living alone, and all-cause mortality: The role of emotional and social loneliness in the elderly during 19 years of follow-up. Psychosom. Med. 81, 521–526. doi: 10.1097/PSY.0000000000000710, PMID: 31094903 PMC6615929

[ref47] Palmonari A. (Ed.). (2011). Psicologia dell’adolescenza [Adolescent Psychology]. Bologna: Il Mulino.

[ref48] Peyser E. (2021). A great excuse to do nothing: The people who don’t want to return to normalcy. *New York Magazine*. Available at: https://nymag.com/intelligencer/2021/04/covid-19-and-the-people-who-dont-want-to-return-to-normalcy.html (Accessed October 20, 2021).

[ref49] Podsakoff P. M. MacKenzie S. B. Lee J. Y. Podsakoff N. P. (2003). Common method biases in behavioral research: A critical review of the literature and recommended remedies. J. Appl. Psychol. 88, 879–903. doi: 10.1037/0021-9010.88.5.879 14516251

[ref50] Robb C. E. de Jager C. A. Ahmadi-Abhari S. Giannakopoulou P. Udeh-Momoh C. McKeand J.. (2020). Associations of social isolation with anxiety and depression during the early COVID-19 pandemic: a survey of older adults in London, UK. Front. Psych. 11:591120. doi: 10.3389/fpsyt.2020.591120, PMID: 33132942 PMC7566017

[ref51] Rokach A. (2015). “Loneliness, alienation, solitude, and our lives,” in Addressing Loneliness. eds. Sha’ked A. Rokach A. (United Kingdom: Psychology Press), 25–41.

[ref52] Rokach A. Brock H. (1998). Coping with loneliness. J. Psychol. 132, 107–127. doi: 10.1080/00223989809599269

[ref53] Rubin K. H. Coplan R. J. Bowker J. C. (2014). On Solitude, Withdrawal, and Social Isolation. Solitude: John Wiley & Sons, Inc.

[ref54] Russell D. W. Cutrona C. E. McRae C. Gomez M. (2012). Is loneliness the same as being alone? J. Psychol. 146, 7–22. doi: 10.1080/00223980.2011.589414, PMID: 22303609

[ref55] Ryan R. M. Deci E. L. (2000a). Self-determination theory and the facilitation of intrinsic motivation, social development, and well-being. Am. Psychol. 55, 68–78. doi: 10.1037/0003-066x.55.1.68 11392867

[ref56] Ryan R. M. Deci E. L. (2000b). Intrinsic and extrinsic motivations: classic definitions and new directions. Contemp. Educ. Psychol. 25, 54–67. doi: 10.1006/ceps.1999.1020 10620381

[ref57] Ryan R. M. Deci E. L. (2017). Self-Determination Theory: Basic Psychological Needs in Motivation, Development, and Wellness. New York: Guilford Publications.

[ref58] Ryan R. M. Deci E. L. Grolnick W. S. (1995). “Autonomy, relatedness, and the self: their relation to development and psychopathology,” in Wiley Series on Personality Processes. Developmental Psychopathology, Vol. 1. Theory and Methods. eds. Cicchetti D. Cohen D. J. (USA: John Wiley & Sons), 618–655.

[ref59] Smith K. J. Victor C. (2019). Typologies of loneliness, living alone and social isolation, and their associations with physical and mental health. Ageing Soc. 39, 1709–1730. doi: 10.1017/S0144686X18000132

[ref60] Thomas V. Azmitia M. (2019). Motivation matters: development and validation of the motivation for solitude scale–short form (MSS-SF). J. Adolesc. 70, 33–42. doi: 10.1016/j.adolescence.2018.11.004, PMID: 30472399

[ref61] Van Baarsen B. Snijders T. A. Smit J. H. Van Duijn M. A. (2001). Lonely but not alone: emotional isolation and social isolation as two distinct dimensions of loneliness in older people. Educ. Psychol. Meas. 61, 119–135. doi: 10.1177/00131640121971103

[ref62] Victor C. Scambler S. Bond J. Bowling A. (2000). Being alone in later life: loneliness, social isolation and living alone. Rev. Clin. Gerontol. 10, 407–417. doi: 10.1017/S0959259800104101

[ref63] Weinstein N. (2014). Human Motivation and Interpersonal Relationships. New York, London: Springer, 1–335.

[ref64] Weinstein N. Hodgins H. S. (2009). The moderating role of autonomy and control on the benefits of written emotion expression. Personal. Soc. Psychol. Bull. 35, 351–364. doi: 10.1177/0146167208328165, PMID: 19223457

[ref65] Weinstein N. Nguyen T. V. (2020). Motivation and preference in isolation: a test of their different influences on responses to self-isolation during the COVID-19 outbreak. R. Soc. Open Sci. 7:200458. doi: 10.1098/rsos.200458, PMID: 32537230 PMC7277280

[ref66] Zessin U. Dickhäuser O. Garbade S. (2015). The relationship between self-compassion and well-being: A meta-analysis. Appl. Psychol. Health Well-Being 7, 340–364. doi: 10.1111/aphw.12051, PMID: 26311196

